# The Foundations of Modern Sociology

**Published:** 1896-06-13

**Authors:** 


### The Foundations of Modern Sociology.

### INTRODUCTION.

The most practical, and some would say the greatest, of the ancient philosophers, Aristotle, begins his work on Ethics with the following words:—

Every art and every kind of inquiry, and likewise every act and purpose, seems to aim at some good; and so it has been well said that the good is that at which everybody aims. But a difference is observable among these aims or ends. What is aimed at is sometimes the exercise of a faculty, sometimes a certain result beyond that exercise. And where there is an end beyond the act, there the result is better than the exercise of the faculty. Now, since there are many kinds of actions and many arts and sciences, it follows that there are many ends also; *e.g.*, health is the end of medicine, ships of shipbuilding, victory of the art of war, and wealth of economy. But when several of these are subordinated to some one art or science—as the making of bridles and other trappings to the art of horsemanship, and this in turn along with all else that the soldier does, to the art of war, and so on—then the end of the master-art is always more desired than the ends of the subordinate arts, since these are pursued for its sake. And this is equally true, whether the end in view be the mere exercise of a faculty or something beyond that, as in the above instances. If, then, in what we do there be some end which we wish for its own account, choosing all the others as means to this, but not every end without exception as a means to something else (for so we should go on *ad infinitum*, and desire would be left void and objectless), this evidently will be the good or the best of all things. And surely from a practical point of view it much concerns us to know this good, for then, like archers shooting at a definite mark, we shall be more likely to attain what we want. If this be so, we must try to indicate roughly what it is, and, first of all, to which of the arts or sciences it belongs. It would seem to belong to the supreme art or science, that one which most of all deserves the name of master art or master science. Now Politics seem to answer to this description, for it prescribes which of the sciences a State needs, and which each man shall study, and up to what point; and to it we see subordinated even the highest arts, such as economy, rhetoric, and the art of war.

We make no apology for the length of this quotation, for probably no other words, either in ancient or modern literature, define so clearly what is meant by the modern word "sociology." To Aristotle "Politics," *i.e.*, the political art or science, meant far more than it does to us, since it concerned the whole field of human life and conduct, making use of all the other political sciences, ordaining both what men are to do and from what they are to refrain; its end including the end of the others, and therefore being the proper good of man. For though the good of the individual is great, that of the State is infinitely greater, and a far grander and more perfect thing both to attain and to secure, and for a number of States greater still, and for the whole of humanity greatest and best of all. Coming back to our own day, perhaps the greatest authorities on "sociology" have been Comte in France, and Buckle and Herbert Spencer in England. More than two thousand years have passed since the days of Aristotle, and he in his own day summarised the results of many ages of observation and experience that had gone before; and yet, in many respects, we seem to be as far off as ever from any definite conclusions regarding what has been called the "sciences of sciences."

We propose, in a short series of article, to trace the growth of the "sociological idea" through those ancient nations which have had the most direct influence upon modern civilisation. Some of them may safely be left out of account, as, for instance, the Egyptians, the Chaldeans, Babylonians, and Assyrians, and the Chinese. Doubtless some of these materially influenced, through the Phœnicians, the later civilisation of Judea, Greece, and Rome. Perhaps even the early civilisation of India may have, in some indirect way, left its impact upon the classical civilisations with which we are most familiar; but this has not yet been clearly proved. After the conquests of Alexander the Great there was, no doubt, a meeting between the Greeks and the inhabitants of India, which for the East, at least, had far-reaching consequences. But when, in the later Roman period, the Romans and the Parthians disputed for the empire of the East, the Greek influence seems to have counted for little, if at all. One solitary historical fact is full of significance. In the year 94 A.D., the Roman legionaries and the soldiers of the Chinese Empire faced each other for a moment from the opposite shores of the Caspian Sea. They never met either in hostile or friendly encounter—and this episode marks very clearly that divergence between the West and the East which has only been partially bridged over in our own times.

The other ancient nations who have most influenced modern civilisation are undoubtedly the Hebrew, the Greek, and the Roman. The inscription on the Cross, which was written in "Hebrew, Greek, and Latin," was an unconscious prophecy by Pontius Pilate. It appealed not only to the ancient but to all the modern world. For, in the Christian world of to-day the morality and the religion is derived from the Hebrew, the philosophy, the arts, and the sciences from the Greek, the grandest conceptions of law and politics from the Roman, who in his own time had chiefly assimilated these ideas from the Orientals and the Greeks who had preceded him, but had also impressed them with the stern stamp of his own individuality.

Our purpose in the ensuing articles will be to endeavour to show how all these various streams of thought and feeling have combined during the progress of the ages, like the tributaries of a great river, to form the one great stream which will eventually lose itself in the ocean of humanity. How the religion and morality of the Hebrew, the intellectuality and art of the Greek, the statesmanship and law of the Roman, have left an indelible impress upon the nations of Europe, who have succeeded to their inheritance and their traditions. The late Professor Freeman, of Oxford, was fond of saying that modern Europe was contained in the two words "Christ and Cæsar," and few students of history judging in a truly impartial spirit will be inclined to doubt the truth of this pregnant remark.

### THE CHISWICK INFECTIOUS HOSPITAL.

The Duke of Devonshire has definitely refused to sell land for a site for an infectious hospital to the Chiswick Urban District Council, on the ground that it would depreciate the value of his own property and that of his tenants.

